# *Pulsatilla* saponin D regulates ras-related C3 botulinum toxin substrate 3 (RAC3) to overcome resistance to paclitaxel in lung adenocarcinoma cells

**DOI:** 10.1186/s12885-024-11841-6

**Published:** 2024-01-10

**Authors:** Yanyan Lu, Wubin He, Xiaoxu Huang, Xuyang Xiao

**Affiliations:** 1 Laboratory Department of Orthopedic Spine Surgery of The First Affiliated Hospital of Jinzhou Medical University, Jinzhou, Liaoning China; 2 Key Laboratory of Surgery of Liaoning Province of The First Affiliated Hospital of Jinzhou Medical University, Jinzhou, Liaoning China; 3 Key Laboratory of Molecular Cell Biology and New Drug Development of Jinzhou Medical University, Jinzhou, Liaoning China; 4 Department of Thoracic Surgery of The First Affiliated Hospital of Jinzhou Medical University, Jinzhou, Liaoning China

**Keywords:** Pulsatilla saponin D, RAC3, Paclitaxel, Resistance, Lung adenocarcinoma cell

## Abstract

**Background:**

Paclitaxel, a tubulin-binding agent, is a Food and Drug Administration-approved first-line drug for the treatment of non-small cell lung cancer (NSCLC), for both squamous and non-squamous cell lung carcinoma, with paclitaxel/carboplatin + bevacizumab a common chemotherapy regimen for stage IV non-squamous NSCLC; however, primary or acquired resistance to paclitaxel is gradually increasing, leading to treatment failure.

**Methods:**

Our results show that Ras-related C3 botulinum toxin substrate 3 (RAC3) is overexpressed in cultured paclitaxel-resistant cells and that *RAC3* expression levels are negatively correlated with sensitivity of lung adenocarcinoma cells to paclitaxel. *Pulsatilla* saponin D could inhibit *RAC3* expression, and we hypothesize that it may block paclitaxel resistance. Further, we found that treatment with paclitaxel combined with *Pulsatilla* saponin D, can overcome lung adenocarcinoma cell resistance to paclitaxel alone in cell culture and mouse xenograft models.

## Introduction

Lung cancer is the malignant tumor that causes the highest morbidity and mortality rates globally, and thus it is a serious threat to human health [1, 2]. Non-small cell lung cancer (NSCLC) accounts for 80–85% of lung cancer incidence [3, 4]. Lung adenocarcinoma, which is the most malignant form of lung cancer, and can develop hematological metastasis at an early stage [5]. Paclitaxel is commonly used for NSCLC chemotherapy. This cytostatic drug blocks mitosis by promoting intracellular microtubule reorganization and inhibiting depolymerization [6]; however, the development of resistance to paclitaxel severely reduces its long-term efficacy [7]. Various mechanisms can cause paclitaxel resistance, including ATP-binding cassette transporter-mediated multidrug resistance, β-tubulin mutations, abnormal expression of apoptotic proteins, cytokine over-expression, and epithelial-mesenchymal transition [8]. PI3K/AKT is over-activated in NSCLC cells [9], and overexpression of AKT1 leads to cisplatin resistance in lung cancer cells, while inhibition of AKT1 expression can reverse multi-drug resistant lung adenocarcinoma cell (A549/CDDP) resistance to cisplatin. Further, AKT1-induced lung cancer cisplatin resistance is mediated via mTOR-P70S6K1 signaling [10, 11].

The dried root of the plant genus, *Pulsatilla* (Family, Ranunculaceae), is used as a traditional Chinese medicine for clearing heat and detoxification [12]. Both total and monomeric *Pulsatilla* saponins have anticancer activity. *Pulsatilla* saponin D (PSD) can inhibit the proliferation of breast [13], pancreatic [14], and lung cancer cells, mostly by inhibiting angiogenesis and by inducing apoptosis [15].

Previous work in our laboratory demonstrated that PSD regulates and inactivates PI3K/AKT signalling and so inhibits the proliferation of lung adenocarcinoma cells. As paclitaxel does not directly act on the PI3K/AKT signaling pathway, we speculated that PSD may act synergistically to enhance the sensitivity of lung adenocarcinoma cells to paclitaxel.

Ras-related C3 botulinum toxin substrate 3 is a member of the RAC subfamily of Rho family proteins that was first reported by Haataja et al. in 1998 [16, 17]. The RAC subfamily includes three main proteins: RAC1, RAC2, and RAC3 [18]. RAC1 is expressed in various tissues, RAC2 is only expressed in the hematopoietic system, and *RAC3* is present in brain and some other tissues [19]. *RAC3* regulates breast cancer invasion and metastasis by controlling adhesion and matrix degradation [20], and promotes proliferation, migration, and invasion in bladder cancer via PYCR1/JAK/STAT signaling [21]. Moreover, endogenous, hyperactive *RAC3* controls breast cancer cell proliferation through a p21-activated kinase-dependent pathway [22].

In our previous study, we found that *RAC3* was overexpressed in A549 paclitaxel-resistant lung adenocarcinoma cells. Therefore, in this study, we investigated whether aberrant *RAC3* expression was involved in the development of adaptive resistance to paclitaxel. The main purpose of our study is to investigate whether PSD affects the expression of *RAC3*, as there are currently no relevant research reports on this topic.

## Materials and methods

### Drugs and reagents

The following reagents were used in the study: Paclitaxel (purity, 99.86%) and Ly294002 (purity, 99.84%) purchased from Selleck Chemical (USA), PSD (purity > 99%) obtained from Nanjing Spring & Autumn Biological Engineering Co(China), MTT and EDU immunofluorescence staining reagent kit from Ribobio (China), Mitochondrial Membrane Potential Detection Kit (JC-1) from Absin (China), 4% paraformaldehyde (PFA), Triton X-100, Hoechst33342, and DAPI from Beyotime (China), TRIzol reagent from Thermo Fisher (USA), BSA from Sigma (USA), and the following antibodies: *RAC3* (Abcam, ab124943), p-PI3K (Affinity, AF3242), and p-AKT (Affinity, AF8355).

### Cell lines and cultures

A549 and NCI-H1299, the human lung adenocarcinoma cell line, was obtained from the Shanghai Institute of Biochemistry and Bell Biology, CAS (China). The cells were cultured in complete DMEM medium, which consisted of 10% fetal bovine serum (USA), 50 µg/mL streptomycin, and 100 U/mL sodium penicillin. The cultures were maintained at 37 °C in a 5% CO2 atmosphere. A549-PR and NCI-H1299-PR cells were derived by treating the A549 and NCI-H1299 cells with paclitaxel at a concentration of 10 nM initially, which was then increased to 40 nM over a period of three months. These A549-PR and NCI-H1299-PR cells were subsequently cultured in complete DMEM with paclitaxel at a concentration of 10 nM to maintain drug resistance in drug-resistant cells. In order to facilitate subsequent experiments, we used the MTT method to detect and confirm the chemotherapy resistance of A549-PR and NCI-H1299-PR cells.

### MTT and EDU immunofluorescence staining assays evaluating cell growth or expansion in vitro

The cells proliferation was evaluated using 3-(4, 5- dimethylthiazol-2-yl)-2, 5- diphenyltetrazolium bromide (MTT) and EDU immunofluorescence staining assays. After the culture with PSD, the cells were incubated with the medium containing 10% MTT solution (5 mg/ mL in PBS) at 37 °C for 4 h. The absorption was measured at 490 nm using a microplate reader (Biotek, Synergy H1/Synergy2, United States).

In the case of EDU immunofluorescence staining, cells were incubated with paclitaxel for 24 h prior to permeabilization. The EDU staining and fixation procedures followed the manufacturer’s instructions. Cell nuclei were stained with DAPI (1 mg/mL, 15 min; Beyotime, China). The numbers of cells were determined using laser scanning confocal microscopy (Olympus, FV10i, Japan).

### Apoptosis

#### Hoechst 33342 staining

Cells in logarithmic growth phase were harvested and inoculated into Petri dishes at a density of 5 × 10^4^ cells per well. The cells were allowed to adhere to the dish surface and establish proper growth conditions. Once the cells had adhered and were exhibiting healthy growth, they were treated with paclitaxel and/or PSD in complete DMEM medium for a duration of 48 h. Following the treatment period, a working solution of Hoechst 33342 was added to each petri dish, ensuring complete coverage of the cells. The dishes were then incubated at 37 °C for 20 min to allow for proper staining. Subsequently, the cells were observed under a fluorescent microscope, and images were captured to document the experimental results.

#### JC-1 staining

Cells in logarithmic growth phase were seeded into culture flasks at a density of 3.5 × 10^4^ cells per milliliter. These cells were then treated with paclitaxel and/or PSD in complete DMEM medium for a duration of 48 h. The solvent control group did not receive any drug treatment. After the 48-hour treatment period, the culture medium was aspirated, and the cells were washed once with PBS. Then, 1 milliliter of culture medium along with the prepared JC-1 working solution was added to the cells. The mixture was thoroughly mixed and incubated at 37 °C for 20 min. Following the incubation, the supernatant was aspirated, and the cells were washed with JC-1 staining buffer 2. Next, fresh culture medium was added to the cells. The cells were then observed under a confocal microscope to determine the ratio of red to green fluorescence. The experimental results were recorded by capturing images during the observation.

### Quantitative real-time PCR detection of the mRNA expression levels of *RAC3* in cells

Total cellular RNA was extracted using TRIzol reagent (Thermo Fisher, USA) according to the manufacturer’s instructions. The mRNA expression levels of *RAC3* were evaluated using SYBR Green qRT-PCR and normalized to the expression levels of *GAPDH*, which served as an endogenous control. The primer sequences used were as follows: *RAC3* F (5’-TCCCCACCGTTTTTGACAACT-3’); *RAC3* R (5’-GCACGAACATTCTCGAAGGAG-3’); *GAPDH* F (5’-TGACTTCAACAGCGACACCCA-3’); *GAPDH* R (5’-CACCCTGTTGCTGAGCCAAAC-3’) (Integrated DNA Technologies, USA).

### Colony formation assay

For the Colony Formation Assay, cells were seeded at a density of 0.5 × 10^3^ cells per well in six-well plates. The cells were then cultured in complete media for a period of 12 days. After the incubation period, the cells were fixed using 4% paraformaldehyde (PFA) in PBS. Subsequently, the fixed cells were stained with 1% crystal violet. The colonies formed by the cells (with a diameter of ≥ 5 mm) were visualized and captured using a digital camera (Olympus, Japan). The number of cell colonies meeting the specified size criterion was counted and recorded.

### Immunofluorescence study

For the Immunoflurescence studies, cells were seeded at a density of 2 × 10^5^ cells per well in culture flasks. After the incubation period, the cells were fixed by adding 4% paraformaldehyde in PBS for 20 min at 37 °C. After fixation, cells were washed three times with PBS to remove excess PFA. To permeabilize fixed cells, cells were treated with 0.1% Triton X-100 in PBS for 10 min at room temperature. Cells were washed 3 times with PBS to remove permeabilization solution. To block nonspecific binding sites, cells were incubated with 1% BSA in PBS for 1 h at room temperature. Dilutions of primary antibodies specific for *RAC3* (1:250), p-PI3K (1:250), and p-AKT (1:250) in antibody dilution buffer (1% BSA in PBS) were prepared. Cells were added the recommended concentration of primary antibody to PBS and incubated overnight at 4℃ with gentle shaking. Cells were washed and appropriate fluorescent secondary antibodies were added at a 1:500 dilution. The dishes were covered the culture flask with aluminum foil, protected from light, and shaked at room temperature for 1 h. Following the treatment period, a working solution of DAPI was added to each petri dish, ensuring complete coverage of the cells. The dishes were then incubated at 37 °C for 20 min to allow for proper staining. Subsequently, the cells were observed under Olymplus Confocal microscope, and images were analysed by ImageJ software.

We acquired immunofluorescence (IF) images using a fluorescence microscope device. Firstly, we ensured good image quality, including appropriate exposure time and resolution. Next, we used the image processing software ‘ImageJ’ to open the IF images and selected areas manually based on shape, size, and location. We employed the fluorescence channel’s measurement tool to measure fluorescence intensity within a selected area, usually a fixed area or the entire cell. For background correction, we measured fluorescence intensity in a cell-free area and subtracted this background value from the measured fluorescence value. This process was repeated multiple times, measuring multiple cells or areas. We then utilized the statistical software ‘GraphPad Prism’ to draw a histogram based on the measured fluorescence intensity values. This allowed us to compare differences in fluorescence signals between different groups for data analysis. Fluorescence Intensity Quantification: Perform processing and analysis of fluorescence images with Image J, which includes procedures such as image enhancement, background correction, and threshold processing. Subsequently, quantitatively calculate the fluorescence intensity for regions or cells of interest, which typically measures the fluorescence intensity value for each region with identical size and shape. Fluorescence Intensity Calculation: Start by selecting regions of interest using a fluorescence microscope and capture the images. Open the images with ImageJ software and select each region with “ROI” (Regions of interest of the same size and shape). Subsequently, employ software to measure the intensity of the fluorescence signal for each selected ROI, processing includes the average grayscale value of fluorescently labeled pixels. Yielding relative fluorescence intensity values for each region by dividing them by the corresponding background fluorescence intensity values (background ROIs).

### Small interfering RNA and plasmids transfection

#### *RAC3* RNA silencing

Cells were plated in 6-well culture plates for 24 h, then transfected with 2 ml transfection medium and 2.5 µl RNAiMAX, along with 2.5 µl siNC (control group) or si*RAC3* (si*RAC3* group). The siRNA sequences used were: siNC F, GCCACGATCCTGACCTAGAG and R ATTCGGCTGAGTCGTACGCC; and si*RAC3* F, GCACGAACATTCTCGAAGGAG and R GCACGAACATTCTCGAAGGAG (Qiagen).

#### *RAC3* overexpression

Cells were plated in 10 cm culture dishes for 24 h, then transfected with with 5 ml transfection medium, containing 25 µl of Lipofectamine 3000 (Invitrogen), and 3 µg of plasmid. Plasmid DNA and Lipofectamine 3000 were diluted in serum-free and antibiotic-free medium. Diluted DNA was added to diluted Lipofectamine 3000, mixed well, and incubated at room temperature for 20 min. RAC3 overexpression group cells were transfected with Flag plasmid, and control group cells were transfected with empty vector. Transfection efficiency was determined by Immunofluorescence staining after 48 h incubation.

### Mouse xenograft assay

Female BALB/c nude mice, aged 6–8 weeks and weighing 18–20 g, were procured from Beijing Vital River Laboratory Animal Technology Co., Ltd (Beijing, China). The mice were housed in a specific pathogen-free (SPF) animal facility under controlled conditions. To establish a subcutaneous gastric tumor model, a total of 5 × 10^6^ A549-PR and NCI-H1299-PR cells were subcutaneously injected near the dorsal flanks of the mice. After two weeks, 20 mice with a tumor volume of 100 mm^3^ were selected and divided into four groups for treatment.

The treatment groups received the following interventions administered once every three days over a 21-day period: DMSO (served as a control, obtained from Selleckchem, USA) with corn germ oil, PSD (30 mg/kg) with corn germ oil, paclitaxel (10 mg/kg) with corn germ oil, or a combination of PSD (30 mg/kg) and paclitaxel (10 mg/kg) with corn germ oil. DMSO and corn germ oil were used as co-solvents for the drugs. The doses mentioned represent the amount of each substance administered per kilogram of the mice’s body weight.

### Data analysis

Data analysis was conducted using GraphPad Prism software version 7.0. Statistical comparisons among groups were performed using one-way ANOVA and chi-square tests to assess single factors. Inter-group analysis was carried out using Student’s t-tests. A significance level of *p* ≤ 0.05 was considered statistically significant.

## Results

### PSD induces apoptosis and inhibits the proliferation of paclitaxel-resistant lung adenocarcinoma cells

To investigate the molecular mechanisms underlying RAC3-associated paclitaxel resistance, we employed a strategy to generate paclitaxel-resistant lung adenocarcinoma cells. These cells were developed through a selection process with low doses of paclitaxel, starting at 10 nM and gradually increasing to 40 nM over a period of three months. Before the experiment, we used the MTT assay to detect the resistance of drug-resistant cells to paclitaxel to ensure the smooth progress of subsequent experiments.

**Fig. 1:**
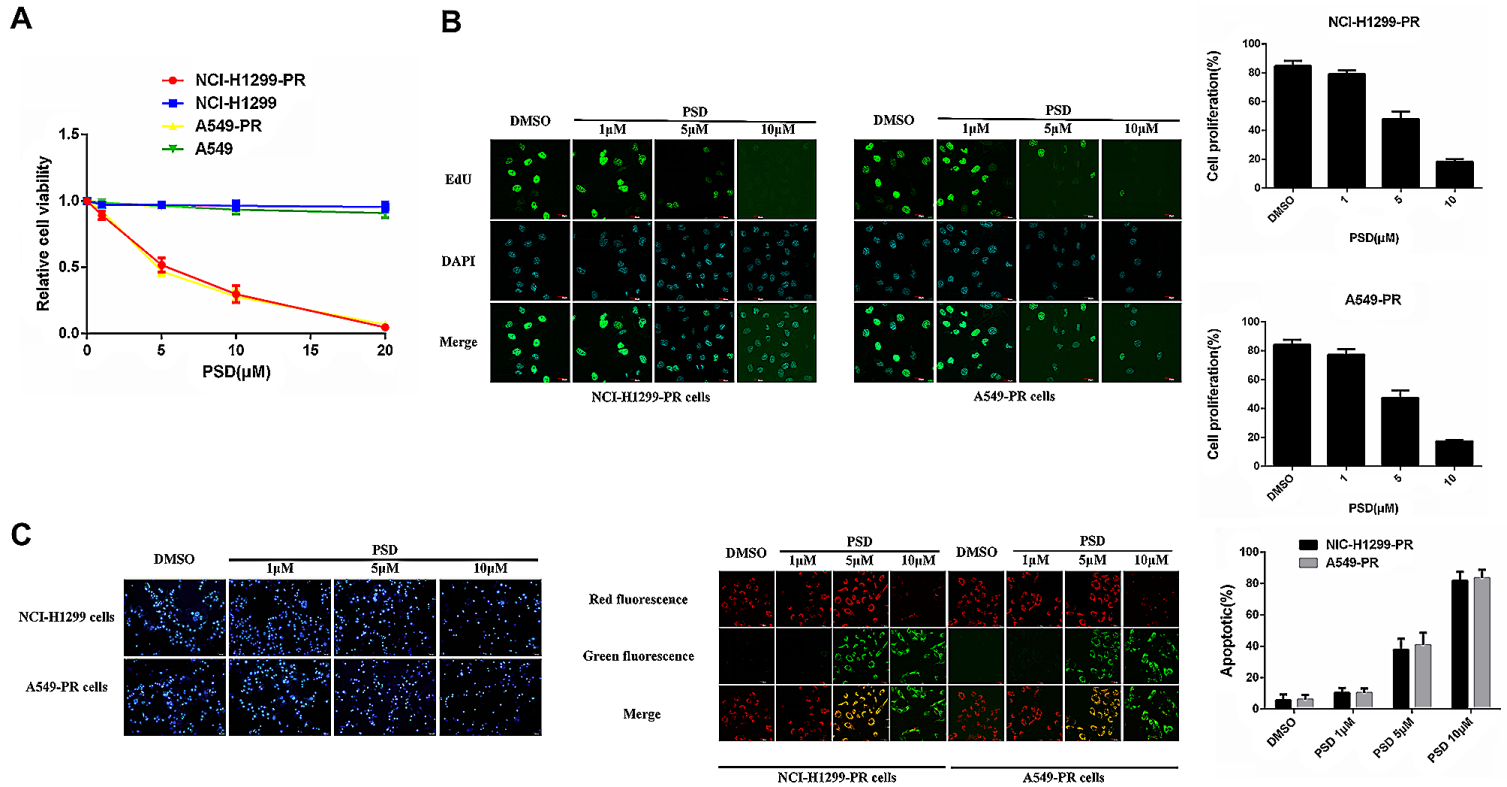
The efficacy of Pulsatilla saponin D (PSD) in inhibiting the proliferation of paclitaxel-resistant cells. (**A**): A549-PR, A549, NCI-H1299 and NCI-H1299-PR cells were subjected to different concentrations of PSD (0, 1, 5, 10, 20 µM), and an MTT assay was conducted after 48 h to evaluate the cells’ sensitivity to PSD. (**B**): Cell proliferation was assessed using an EDU assay. The cells were stained with DAPI (blue) to visualize the nuclei, and proliferating cells were identified by the presence of green fluorescence (EDU). The number of EDU-positive cells per field was quantified from multiple fields, and cell proliferation was calculated relative to the total cell count. Scale bars represent 20 μm. (**C**): Apoptosis was detected through Hoechst 33,342 and JC-1 staining. The nuclei were stained blue with Hoechst 33,342. Apoptotic changes were indicated by increased fluorescence intensity, nuclear pyknosis, and fragmentation. The JC-1 staining assessed apoptosis by measuring the mitochondrial membrane potential. In viable cells, JC-1 aggregated in the mitochondrial matrix, producing red fluorescence. However, in early apoptotic cells, JC-1 existed as monomers and emitted green fluorescence. The ratio of green-stained cells to the total cell count was quantified from multiple fields. Scale bars: Hoechst 33,342 staining (100 μm), JC-1 staining (20 μm). The experiments were repeated three times

The results showed that PSD treatment alone had a dose-dependent inhibitory effect on the proliferation of A549-PR and NCI-H1299-PR lung adenocarcinoma cells, but had no obvious inhibitory effect on the proliferation of sensitive A549 and NCI-H1299 cells. (Fig. 1A). To further evaluate the effect of PSD, we performed a 5-ethynyl-2’-deoxyuridine (EDU) immunofluorescence staining assay to assess cell viability after 24 h of treatment. The findings indicated that PSD treatment at various concentrations effectively suppressed the growth of both A549-PR and NCI-H1299-PR cells (Fig. 1B).

Moreover, we employed Hoechst 33,342 and JC-1 staining apoptosis assays to investigate the apoptotic response of A549-PR and NCI-H1299-PR cells to PSD treatment. The results revealed that PSD induced dose-dependent apoptosis in both cell types (Fig. 1C). These findings shed light on the potential of PSD to induce apoptosis and inhibit the proliferation of paclitaxel-resistant A549-PR and NCI-H1299-PR cells.

### PSD inhibits RAC3 expression in paclitaxel-resistant lung adenocarcinoma cells

**Fig. 2:**
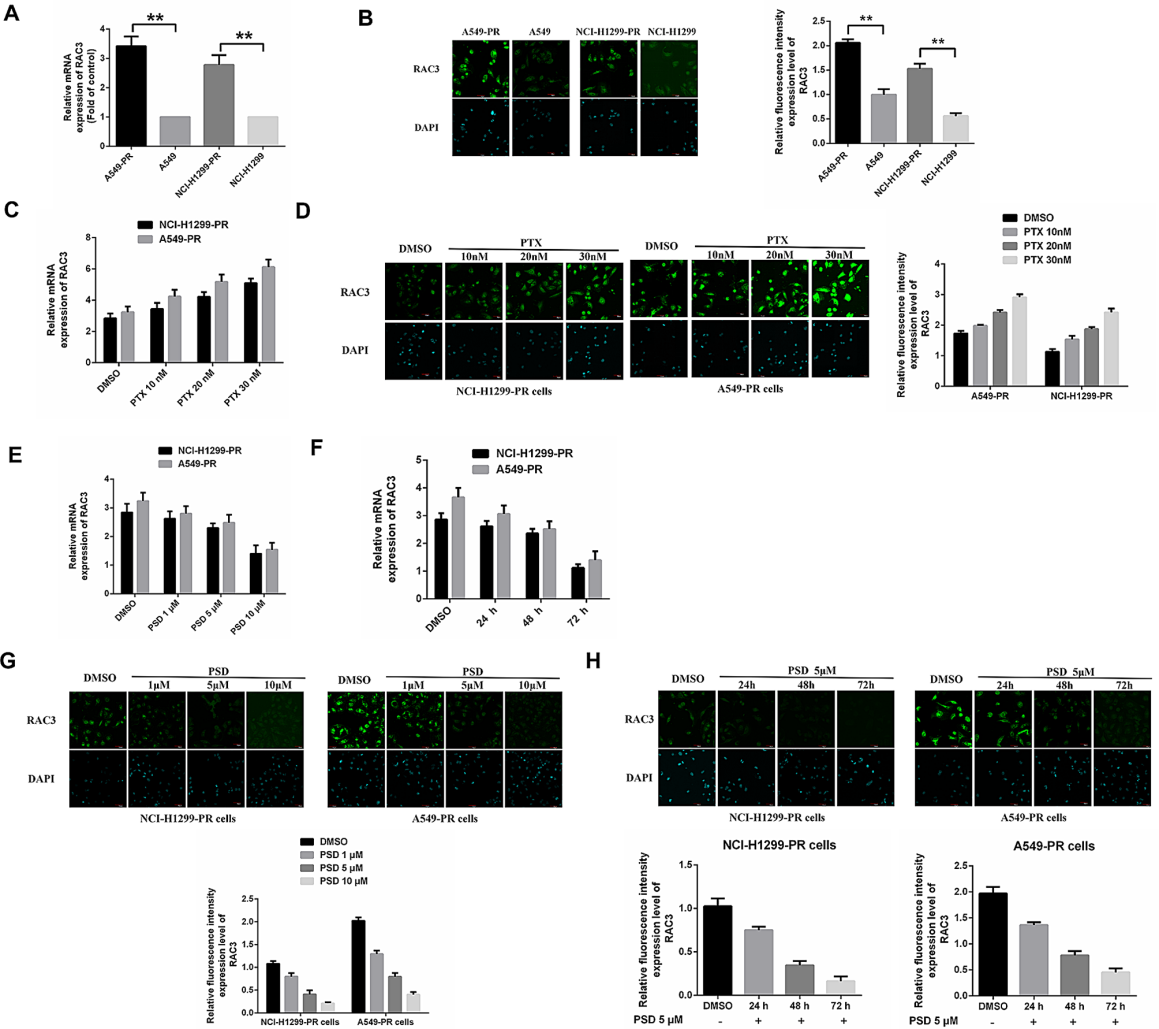
Pulsatilla saponin D (PSD) exhibits inhibitory effects on *RAC3* expression in paclitaxel-resistant cells. (**A-B**): The elevated expression of *RAC3* in A549-PR and NCI-H1299-PR cells was confirmed through:(A) qRT-PCR analysis and (B) Immunofluorescence staining. (**C-D**): Immunofluorescence staining and qRT-PCR analysis demonstrated that *RAC3* expression in A549-PR and NCI-H1299-PR cells increased in a dose-dependent manner upon exposure to paclitaxel. This experiment was conducted in triplicate. (**E-F**): qRT-PCR analysis revealed that PSD effectively suppressed *RAC3* mRNA expression in A549-PR and NCI-H1299-PR cells, in a dose-dependent (E) and time-dependent (F) manner. (**G-H**): Immunofluorescence staining also revealed that PSD effectively suppressed *RAC3* expression in A549-PR and NCI-H1299-PR cells, in a dose-dependent (G) and time-dependent (H) manner. The scale bar for immunofluorescence staining was 50 μm. The experiments were repeated three times, and the data are presented as mean ± standard deviation (SD). Statistical significance (*P < 0.05) was determined using Student’s t-test

We proceeded to investigate the expression of the *RAC3* gene in paclitaxel-resistant lung adenocarcinoma cells and examined whether its levels were affected by treatment with paclitaxel or PSD. Both quantitative real-time polymerase chain reaction (qRT-PCR) and immunofluorescence staining analysis were employed to assess *RAC3* expression. The results revealed significantly higher levels of *RAC3* expression in paclitaxel-resistant cells compared to non-resistant cells (Fig. 2A, B). Additionally, the expression of *RAC3* in paclitaxel-resistant cells was found to be influenced by paclitaxel treatment in a dose-dependent manner (Fig. 2C, D).

Given the inhibitory effect of PSD on the proliferation of paclitaxel-resistant lung adenocarcinoma cells, we hypothesized that PSD might also impact *RAC3* expression in this cellular context. To test this hypothesis, paclitaxel-resistant cells were treated with various concentrations of PSD for different durations. The results demonstrated that PSD treatment dose-dependently reduced the expression of *RAC3* in paclitaxel-resistant cells (Fig. 2E, G). Moreover, the inhibitory effect of PSD on *RAC3* expression was observed to be time-dependent (Fig. 2F, H).

### RAC3 knockdown can restore the sensitivity of paclitaxel-resistant lung adenocarcinoma cells to paclitaxel

**Fig. 3:**
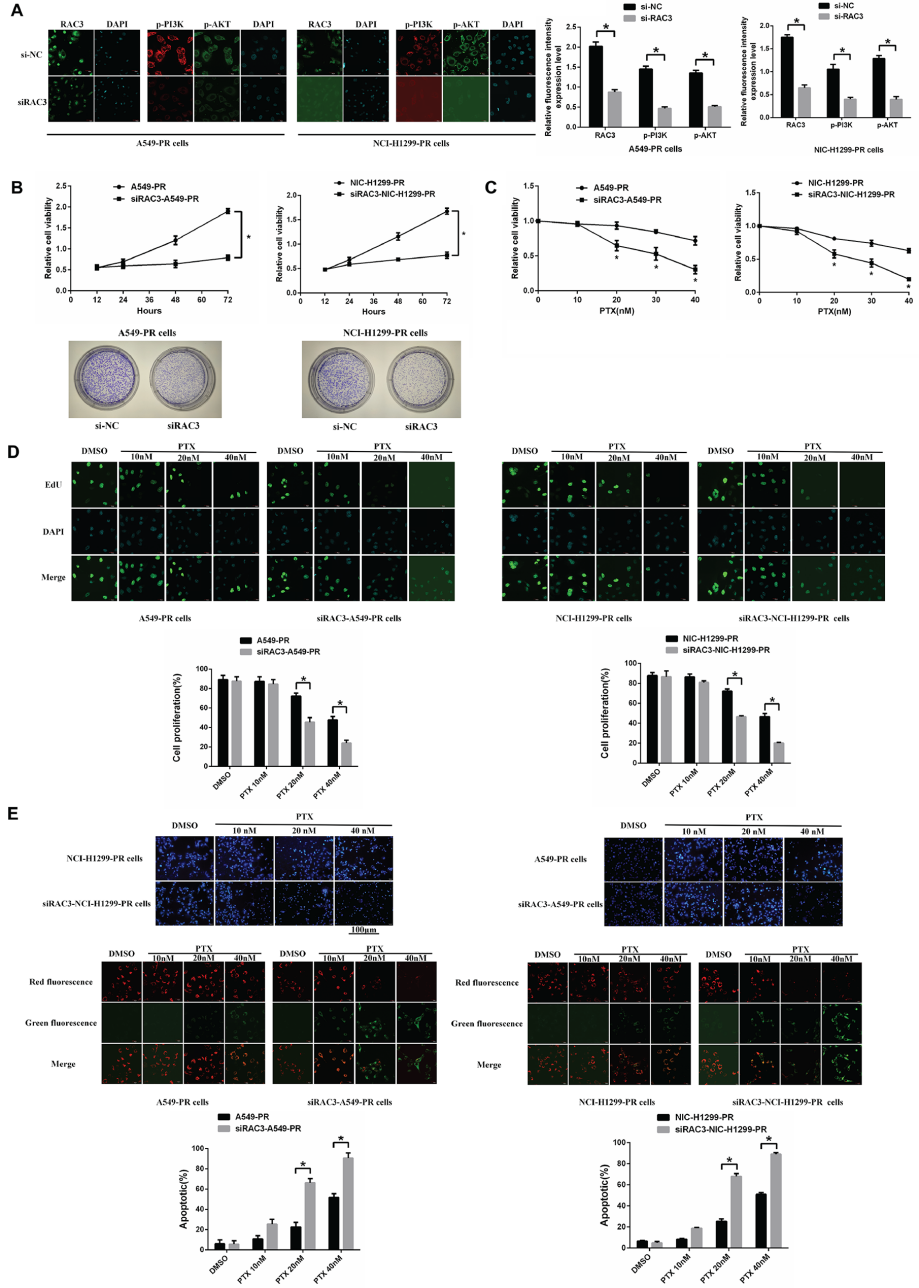
*RAC3* knockdown reduces paclitaxel resistance. (**A**): Immunofluorescence staining confirms successful *RAC3* knockdown. Scale bars: *RAC3* staining, 50 μm; p-PI3K and p-AKT staining, 20 μm. (**B**): Growth of *RAC3* knockdown and normal control A549-PR and NCI-H1299-PR cells assessed by MTT assay (72 h) and colony formation assay (12 days). Data are presented as mean ± standard deviation (SD). (**C**): Sensitivity of A549-PR and NCI-H1299-PR cells to different concentrations of paclitaxel (0, 10, 20, 30, or 40 nM) determined by MTT assay after 48 h. (**D**): Cell proliferation evaluated by EDU assay. Scale bars, 20 μm. (**E**): Apoptosis detected through Hoechst 33,342 and JC-1 staining. Scale bars: Hoechst 33,342 staining, 100 μm; JC-1 staining, 20 μm.The experiments were repeated three times, and the data are presented as mean ± standard deviation (SD). Statistical significance (*P < 0.05) was determined using Student’s t-test

In order to determine the role of *RAC3* in paclitaxel resistance, we employed RNA interference to knock down the expression of *RAC3* in A549-PR and NCI-H1299-PR cells. The effectiveness of the knockdown was confirmed through immunofluorescence staining, which showed reduced *RAC3* levels. At the same time, we observed that low expression of *RAC3* inhibited the phosphorylation levels of PI3K and AKT in paclitaxel-resistant lung adenocarcinoma cells (Fig. 3A). Subsequently, MTT and colony formation assays were conducted, revealing that the downregulation of *RAC3* resulted in decreased viability of A549-PR and NCI-H1299-PR cells (Fig. 3B). Similarly, MTT and EDU immunofluorescence staining assays were performed after treatment with different concentrations of paclitaxel. These experiments demonstrated that the knockdown of *RAC3* in A549-PR cells significantly reduced cell proliferation, particularly when combined with paclitaxel treatment (Fig. 3C, D). Additionally, Hoechst 33,342 and JC-1 staining apoptosis assays showed that *RAC3*-knockdown cells exhibited significantly higher levels of apoptosis than mock-treated control cells, particularly when exposed to a low concentration of paclitaxel (20 nM) (Fig. 3E).

### RAC3 overexpression in lung adenocarcinoma cells promotes paclitaxel resistance

**Fig. 4:**
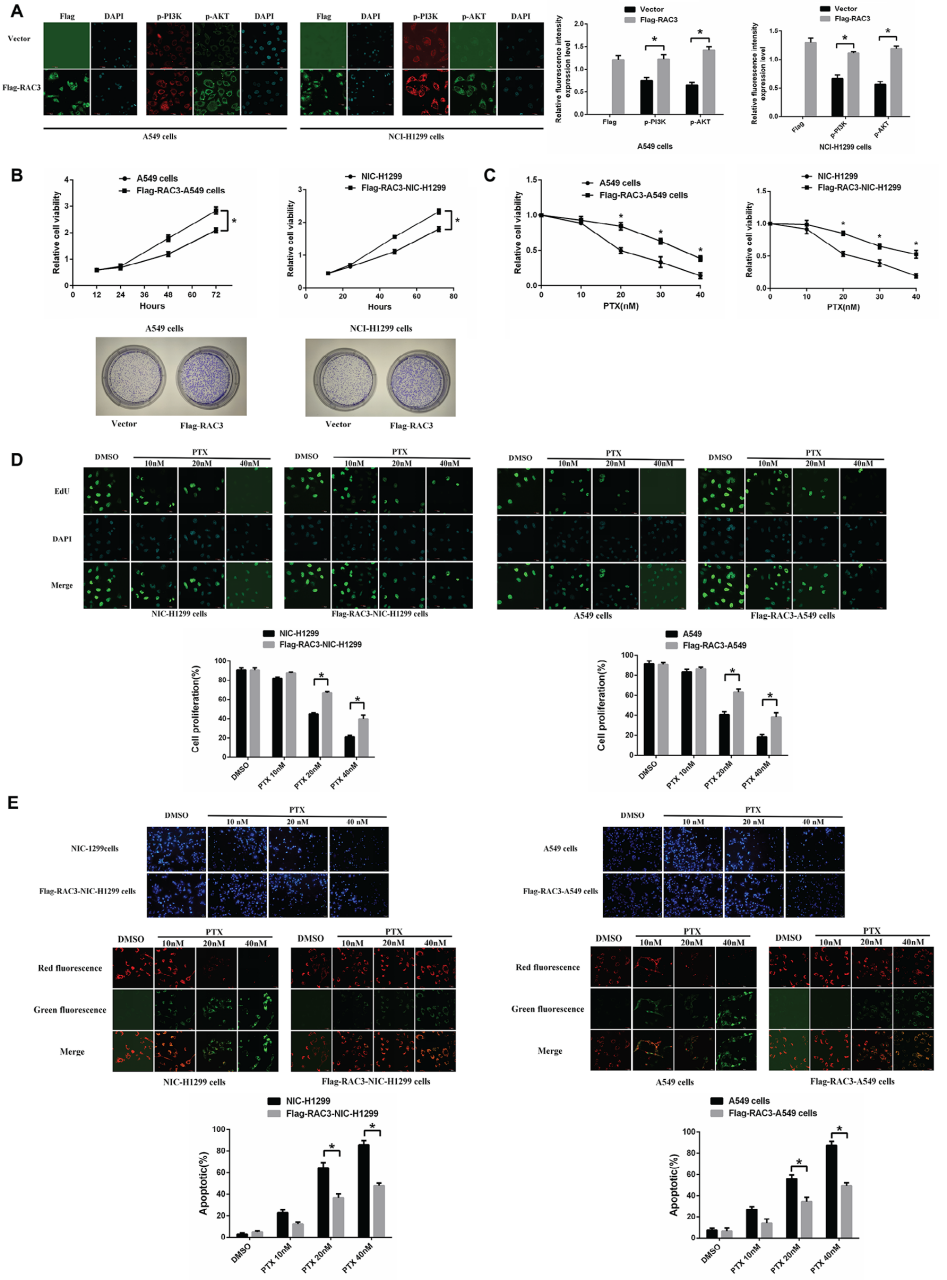
Overexpression of *RAC3* enhances lung adenocarcinoma cell resistance to paclitaxel. (**A**): Immunofluorescence staining confirms successful overexpression of *RAC3*. Scale bars: *RAC3* staining, 50 μm; p-PI3K and p-AKT staining, 20 μm. (**B**): Growth of *RAC3*-overexpressing and normal control A549 and NCI-H1299 cells assessed by MTT assay (72 h) and colony formation assay (12 days). Data are presented as mean ± standard deviation (SD). (**C**): Paclitaxel sensitivity of A549 cells evaluated by MTT assay after 48 h. (**D**): Cell proliferation measured by EDU assay. Scale bars, 20 μm. (**E**): Apoptosis detected through Hoechst 33,342 and JC-1 staining. Scale bars: Hoechst 33,342 staining, 100 μm; JC-1 staining, 20 μm.The experiments were repeated three times, and the data are presented as mean ± SD. Statistical significance (*P < 0.05) was determined using Student’s t-test

To assess the role of *RAC3* in paclitaxel resistance, we conducted an experiment where we overexpressed *RAC3* in parental A549 and NCI-H1299 cells. The successful overexpression of *RAC3* was clearly confirmed (Fig. 4A). Moreover, we observed that overexpression of *RAC3* promoted the phosphorylation levels of PI3K and AKT in parental A549 and NCI-H1299 cells. Subsequently, we performed MTT assays at a 72-hour time point and colony formation assays over a 14-day period, which demonstrated that *RAC3* overexpression in A549 and NCI-H1299 cells led to increased cell viability (Fig. 4B). Moreover, our findings revealed that the overexpression of *RAC3* in A549 and NCI-H1299 cells significantly decreased their sensitivity to paclitaxel, as observed in Fig. 4C and D. Additionally, we observed reduced rates of apoptosis in *RAC3*-overexpressing cells following paclitaxel treatment (Fig. 4E).

### PSD combined with paclitaxel overcomes adaptive resistance to paclitaxel in vivo and in vitro

PSD inhibits *RAC3* expression in paclitaxel-resistant lung adenocarcinoma cells. Based on this observation, we formulated a hypothesis that PSD, in combination with paclitaxel, could be utilized to downregulate *RAC3* and inhibit paclitaxel resistance in lung adenocarcinoma cells.

**Fig. 5:**
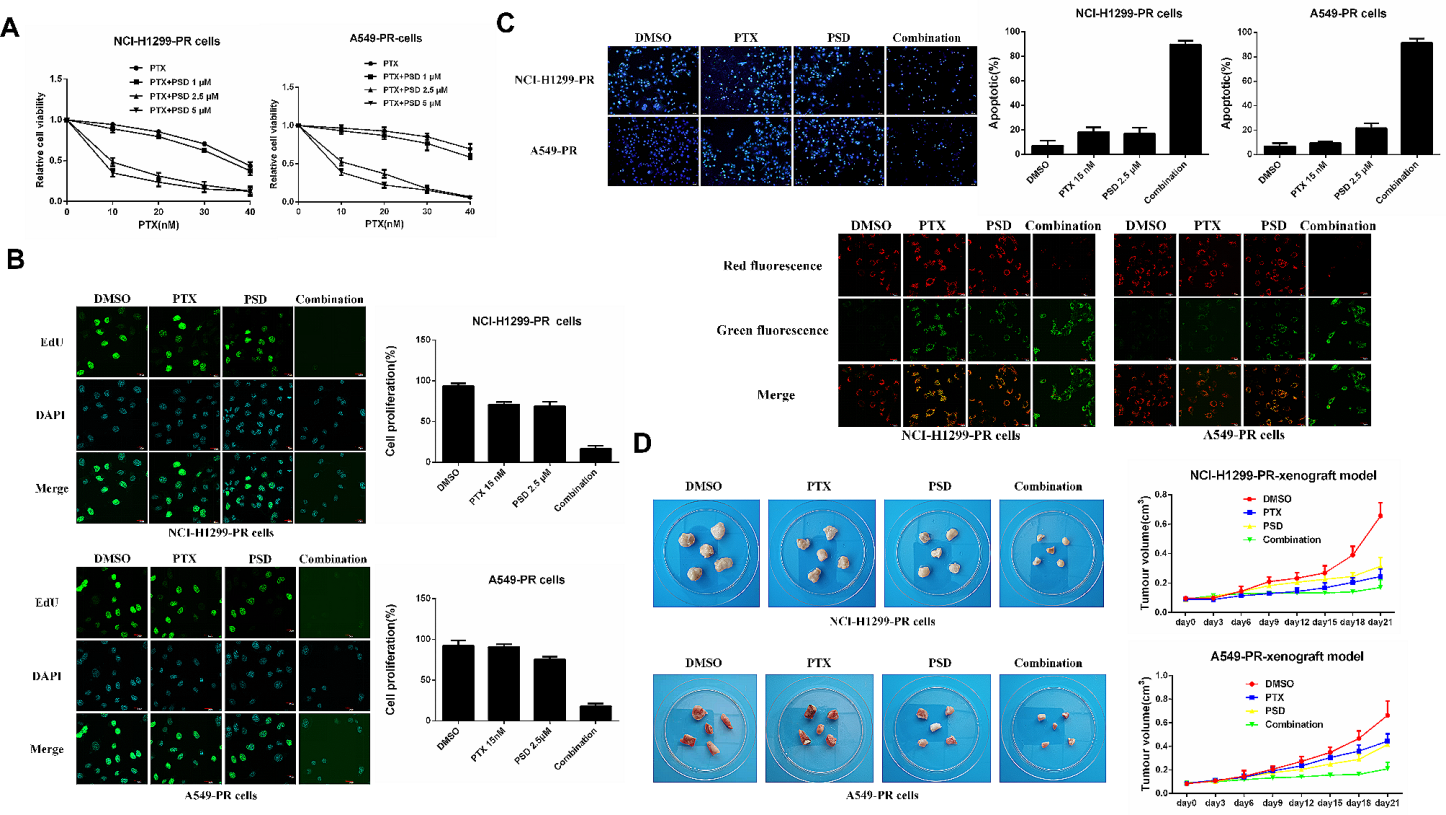
Combination of Pulsatilla saponin D (PSD) and paclitaxel overcomes adaptive resistance to paclitaxel both in vivo and in vitro. (**A**): MTT assays were performed after 48 h to evaluate the efficacy of paclitaxel alone or in combination with a fixed dose of PSD. (**B**): Cell proliferation was assessed by EDU assay after 24 h of treatment with paclitaxel and PSD alone or in combination in A549-PR and NCI-H1299-PR cells. Scale bars, 20 μm. (**C**): Apoptosis was detected by Hoechst 33,342 and JC-1 staining in A549-PR and A549 cells treated with paclitaxel and PSD alone or in combination for 48 h. Scale bars: Hoechst 33,342 staining, 100 μm; JC-1 staining, 20 μm. (**D**): Xenograft tumor models were established by injecting approximately 5 × 10^6^ A549-PR and NCI-H1299-PR cells into mouse flanks. Once tumors reached a size of ≥ 100 mm^3^, mice were treated with the following regimens: (1) DMSO with corn germ oil; (2) Paclitaxel (10 mg/kg) with corn germ oil; (3) PSD (30 mg/kg) with corn germ oil; and (4) Paclitaxel (10 mg/kg) and PSD (30 mg/kg) with corn germ oil. Treatments were administered once every three days via oral cannula administration. Tumor size was measured periodically using calipers, and tumor volumes were calculated using the formula: volume = width^2^ × length/2

To investigate this hypothesis, we conducted both in vitro and in vivo studies to evaluate the inhibitory effects of PSD alone and in combination with paclitaxel on paclitaxel-resistant lung adenocarcinoma cells. Initially, we determined the appropriate concentrations of paclitaxel and PSD for single-agent treatment and combination therapy using an MTT assay (Fig. 5A). Subsequently, we selected concentrations of 15 nM paclitaxel and 2.5 µM PSD for the combination treatment of A549-PR and NCI-H1299-PR cells. After 48 h of treatment, we observed that PSD significantly enhanced the sensitivity of lung adenocarcinoma cells to paclitaxel, resulting in inhibited proliferation and increased apoptosis, which are indicative of improved paclitaxel sensitivity (Fig. 5B, C).

Furthermore, we employed xenograft models established from A549-PR and NCI-H1299-PR cells to examine the efficacy of the treatments in vivo. Once the xenograft tumors reached a volume of ≥ 100 mm^3^, the animals were divided into four groups: (1) dimethyl sulfoxide (DMSO) with corn germ oil, (2) paclitaxel (30 mg/kg) with corn germ oil, (3) PSD (50 mg/kg) with corn germ oil, and (4) paclitaxel (30 mg/kg) and PSD (50 mg/kg) with corn germ oil. After a 3-week treatment period, we observed inhibited cancer growth, as indicated by reduced tumor weight, in animals treated with either paclitaxel or PSD alone. Notably, the combination treatment of PSD and paclitaxel resulted in further growth inhibition compared to the individual treatments in both A549-PR and NCI-H1299-PR xenograft tumors (Fig. 5D).

In summary, our findings from in vitro and in vivo studies support the hypothesis that PSD, when used in combination with paclitaxel, can effectively downregulate *RAC3* expression and inhibit paclitaxel resistance in lung adenocarcinoma cells.

### PSD blocks RAC3-induced paclitaxel resistance via suppressing the PI3K/AKT pathway

**Fig. 6:**
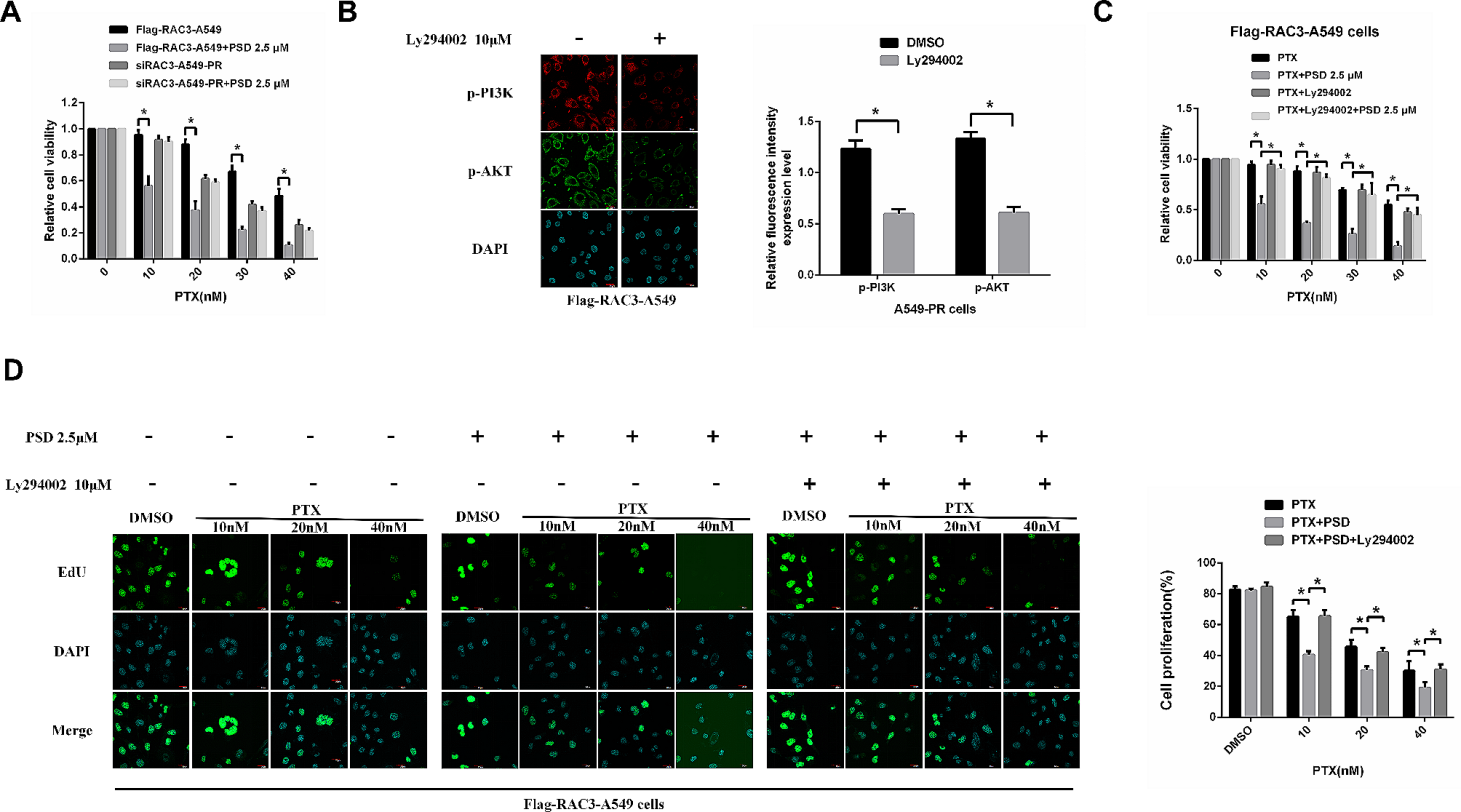
Suppression of *RAC3*-induced paclitaxel resistance by Pulsatilla saponin D (PSD) through inhibition of the PI3K/AKT pathway. (**A**): MTT assay was performed to evaluate cell proliferation in A549-PR cells with *RAC3* downregulation and A549 cells with *RAC3* overexpression, treated with paclitaxel alone or in combination with PSD. (**B**): Immunofluorescence staining was used to assess the levels of phosphorylated PI3K (p-PI3K) and AKT (p-AKT) in A549 cells with *RAC3* overexpression. Treatment with 10 µM Ly294002 for 7 days inhibited p-PI3K and p-AKT levels, as determined by immunofluorescence staining. Scale bars, 20 μm. (**C**): MTT assay was conducted to evaluate the effect of PSD-induced paclitaxel sensitization in A549 cells overexpressing *RAC3*, treated with 10 µM Ly294002 for 7 days. (**D**): EDU assay was performed to determine the proliferation of A549 cells overexpressing *RAC3*, treated with 10 µM Ly294002 for 7 days. Scale bars, 20 μm. Experiments were repeated three times and data are presented as mean ± standard deviation (SD). *P < 0.05 (Student’s t-test)

To gain further insights into the impact of *RAC3* downregulation and overexpression on PSD-induced paclitaxel sensitization, we conducted experiments to determine the appropriate concentration of paclitaxel, alone or in combination with PSD, in A549-PR and NCI-H1299-PR cells with *RAC3* downregulated and A549 and NCI-H1299 cells overexpressing *RAC3*. This was assessed using an MTT assay (Fig. 6A). Our findings revealed that PSD effectively inhibited cell proliferation only in cells overexpressing *RAC3*.

The above results suggest that RAC3/PI3K/AKT may be involved in the resistance of lung adenocarcinoma cells to paclitaxel. PSD inhibits *RAC3* expression in paclitaxel-resistant lung adenocarcinoma cells. We propose that PSD may increase the sensitivity of lung adenocarcinoma cells to PTX by inhibiting RAC3/PI3K/AKT. To validate the notion that *RAC3* promotes paclitaxel-resistant lung adenocarcinoma cell proliferation and survival through the activation of PI3K/AKT signaling, we treated A549 cells overexpressing *RAC3* with 10 µM Ly294002, an inhibitor of PI3K. After 7 days of treatment, the results of immunofluorescence staining indicated that Ly294002 effectively inhibited the levels of p-PI3K and p-AKT in A549 cells overexpressing *RAC3* (Fig. 6B). Additionally, MTT and EDU immunofluorescence staining assays conducted over a 24-hour period demonstrated that the paclitaxel sensitization induced by PSD was significantly diminished in A549 cells overexpressing *RAC3* compared to Ly294002-untreated cells (Fig. 6C, D).

In summary, our experiments provided evidence that PSD-induced paclitaxel sensitization is influenced by the modulation of *RAC3* expression. PSD effectively inhibits cell proliferation specifically in cells overexpressing *RAC3*. Furthermore, the activation of PI3K/AKT signaling, promoted by *RAC3*, contributes to A549 cell proliferation and survival. This is substantiated by the inhibitory effects of Ly294002 on p-PI3K and p-AKT levels, as well as the attenuation of PSD-induced paclitaxel sensitization in A549 cells overexpressing *RAC3*.

## Discussion

Paclitaxel is the main active component of Taxus brevifolia extract and it acts as a microtubule stabilizer [23]; It promotes the polymerization of tubulin to form microtubule structures in tumour cells, and then interferes with the cell cycle and mitosis, which leads to cell death [24]. Drug resistance is an important factor affecting the efficacy of paclitaxel chemotherapy [25]. PSD is a widely-used effective anti-malarial agent, that also has anti-tumor effects [26]. Our in vitro experimental results show that PSD can significantly inhibit the proliferation of drug-resistant lung adenocarcinoma cells compared with sensitive lung adenocarcinoma cells. PSD reduces cell proliferation and promotes apoptosis in a dose-dependent manner.

*RAC3* is an important co-activator of androgen receptor, in the prostate, and it may have a significant role in prostate cancer progression [27]. Studies investigating the nuclear receptor coactivator, *RAC3*, indicate that it functions in many biological processes and tumorigenesis, it has a clear role in the development of resistance to chemotherapy in colon cancer cells through autophagy and apoptosis inhibition [28]. *RAC3* was over-expressed in human chronic myeloid leukemia K562 cells, which are normally resistant to TRAIL-induced apoptosis [29]. However, whether *RAC3* can affect the development of paclitaxel resistance has not been reported.

In this study, paclitaxel-resistant lung cancer cells showed strong proliferation and reduced apoptosis in the presence of paclitaxel. Additionally, *RAC3* was overexpressed in paclitaxel-resistant cells, and paclitaxel promoted *RAC3* expression in paclitaxel-resistant lung cancer cells in a dose-dependent fashion. At the same time, we found that PSD can significantly inhibit the expression of *RAC3* in paclitaxel-resistant lung adenocarcinoma cells, and the inhibitory effect is time- and concentration-dependent. Therefore, we believe that PSD treatment alone has a dose-dependent inhibitory effect on the proliferation of A549-PR and NCI-H1299-PR lung adenocarcinoma cells, but has no obvious inhibitory effect on the proliferation of sensitive A549 and NCI-H1299 cells. The reason is that the expression of *RAC3* in A549-PR and NCI-H1299-PR cells is significantly higher than that in sensitive A549 and NCI-H1299 cells. This suggests that PSD may inhibit the proliferation of paclitaxel-resistant lung adenocarcinoma cells by reducing *RAC3* expression.

To test whether *RAC3* is involved in the development of paclitaxel resistance in lung adenocarcinoma cells, we increased the expression of *RAC3* in sensitive cells and decreased the expression of *RAC3* in resistant cells. The results show that *RAC3* knockout A549-PR cells have increased sensitivity to paclitaxel, and the resistance of paclitaxel-sensitive lung adenocarcinoma cells to paclitaxel can be explained by the overexpression of *RAC3*. We concluded that *RAC3* expression in A549-PR cells is associated with paclitaxel resistance.

PSD may inhibit the proliferation of paclitaxel-resistant lung adenocarcinoma cells by reducing *RAC3* expression. On the base of the data, we suggested that, in combination with paclitaxel, PSD can increase lung adenocarcinoma cell sensitivity to paclitaxel. This study also confirmed that PSD combined with paclitaxel overcomes adaptive resistance to paclitaxel in vivo and in vitro. A549 cells overexpressing *RAC3* and A549-PR cells with downregulated *RAC3* were treated with different concentrations of paclitaxel alone or in combination with PSD. We found that PSD could only effectively inhibit cell proliferation in *RAC3* overexpressing cells, indicating that PSD enhances the sensitivity of lung cancer cells to paclitaxel by inhibiting *RAC3* expression.

PSD not only induced apoptosis, but also inhibited cell growth and angiogenesis, through modulation of the PI3K/Akt/mTOR pathway in human hepatocellular carcinoma [30]. Further, PSD inhibits the AKT/mTOR pathway, leading to the suppression of colon tumour growth and angiogenesis, together with induction of apoptosis [31]. In our study, we examined the PI3K/AKT pathway using immunofluorescence staining. *RAC3* expression was positively correlated with activation of phosphorylated PI3K, confirming that the PI3K/AKT pathway is significantly upregulated in A549-PR and NCI-H1299-PR cells. As paclitaxel does not directly act on the PI3K/AKT signalling pathway, we speculated that PSD may act synergistically to enhance the sensitivity of lung adenocarcinoma cells to paclitaxel. In order to prove this conjecture, we used Ly294002, a highly selective PI3K inhibitor, was used to treat *RAC3*-overexpressing A549 cells for 7 days. Compared to the non-treated Ly294002 group, PSD-induced paclitaxel sensitivity was reduced by the PI3K inhibitor. This result indicates that *RAC3* promotes A549 cell proliferation and survival through PI3K/AKT pathway activation, and that PSD can overcome *RAC3*-induced paclitaxel resistance by suppressing the PI3K/AKT pathway.

The main limitation of this study is how *RAC3* activates the PI3K/AKT signaling pathway, thereby increasing the sensitivity of lung cancer cells to paclitaxel. The specific mechanism still needs further study.

## Conclusion

Together, the results of the current study demonstrate that *RAC3* is highly expressed in paclitaxel-resistant lung adenocarcinoma cancer cells, and is a functional marker that is essential for paclitaxel resistance via activation of the PI3K/AKT pathway. PSD can increase lung cancer cell sensitivity to paclitaxel via inhibition of *RAC3*.

## Data Availability

The raw data supporting the conclusions of this article will be made available by the authors, without undue reservation, to any qualified researcher. Xuyang Xiao should be contacted if someone wants to request the data from this study.
